# Supplementary material for: Identification of photoperiod-induced specific miRNAs in the adrenal glands of Sunite sheep (Ovis aries)
Source: Front Vet Sci. 2022 Jul 22;9:888207. doi: 10.3389/fvets.2022.888207 (PMC9354845; doi:10.3389/fvets.2022.888207)
Supplement: Supplementary file 1 [file Data_Sheet_1.ZIP › Supplementary file/miRDeep2 file.zip!/miRDeep2 file/Novel_Structure/SPLP42A2/Novel_186.pdf]

Provisional ID	:	NC_019467.2_21048
Score total	:	2.1
Score for star read(s)	:	-1.3
Score for read counts	:	0
Score for mfe	:	1.8
Score for randfold	:	1.6
Score for cons. seed	:	
Total read count	:	135
Mature read count	:	135
Loop read count	:	0
Star read count	:	0

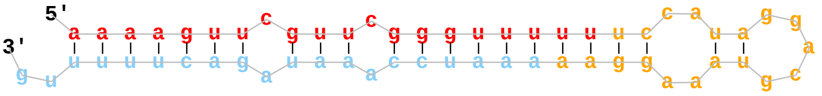

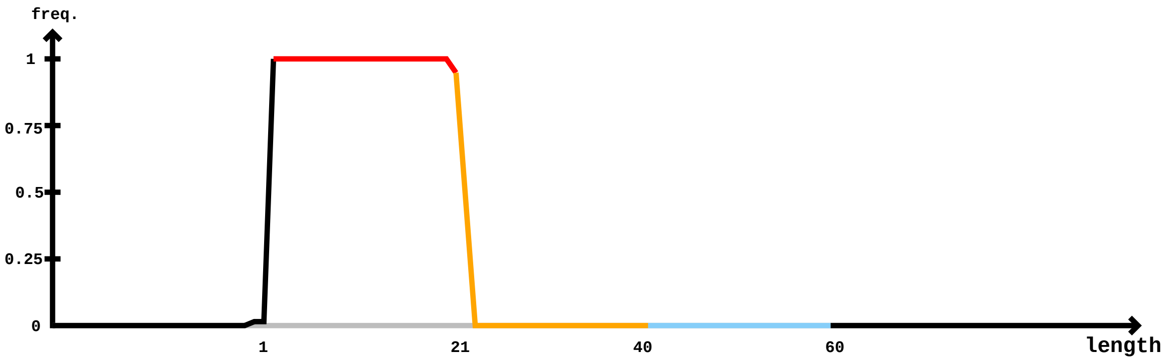

Mature **Star**

```
5'- uugaaaguauuggguuggcccaaaaguucguucggguuuuuuccauaggacguaaaggaaaaauccaaauagacuuuuuggccaacccaaucaauauauguugaugucccu   -3'    exp
    .........(((((((((.(((((((.(((.((((((((((..((.....))..)))))))))).))).))))))).))))))))))((((((....))))))......    reads      mm        sample
    ...................cAaaaaguucguucggguuuuu......................................................................    2          1         seq
    .....................aaaaguucguucggguuuu.......................................................................    7          0         seq
    .....................aaaaguucguucggguuuuu......................................................................    126        0         seq
```
